# Applying HPLC to Screening QTLs for BLB Resistance in Rice

**DOI:** 10.3390/plants10102145

**Published:** 2021-10-09

**Authors:** Xiao-Xuan Du, Jae-Ryoung Park, Xiao-Han Wang, Yoon-Hee Jang, Eun-Gyeong Kim, Gang-Seob Lee, Kyung-Min Kim

**Affiliations:** 1 Biosafety Division, National Academy of Agricultural Science, Rural Development Administration, Jeonju 54874, Korea; haobingshuaike@hotmail.com; 2 Coastal Agriculture Research Institute, Kyungpook National University, Daegu 41566, Korea; icd92@naver.com; 3 Division of Plant Biosciences, School of Applied Biosciences, College of Agriculture and Life Science, Kyungpook National University, Daegu 41566, Korea; uniunnie@naver.com (Y.-H.J.); dkqkxk632@naver.com (E.-G.K.); 4 National Agrobiodiversity Center, National Institute of Agricultural Sciences, Rural Development Administration, Jeonju 55365, Korea; wang@knu.ac.kr

**Keywords:** HPLC, QTL, rice, BLB, *Xanthomonas oryzae* pv. *oryzae*

## Abstract

Bacterial leaf blight (BLB) is caused by *Xanthomonas oryzae* pv. *oryzae* and is a major cause of rice yield reductions around the world. When diseased, plants produce a variety of metabolites to resist pathogens. In this study, the various defense metabolites were quantified using high-performance liquid chromatography (HPLC) after Xoo inoculation in a 120 Cheongcheong/Nagdong double haploid (CNDH) population. Quantitative trait locus (QTL) mapping was conducted using the concentration of the plant defense metabolites. HPLC analyzes the concentration of substances according to the severity of disease symptoms. Searching for BLB resistance candidate genes by applying this analysis method is very effective when mapping related genes. These resistance genes can be mapped directly to the causative pathogens. A total of 17 metabolites were detected by means of HPLC analysis after Xoo inoculation in the 120 CNDH population. QTL mapping of the metabolite concentrations resulted in the detection of the BLB resistance candidate gene, *OsWRKYq6,* in RM3343 of chromosome 6. *OsWRKYq6* has a very high homology sequence with WRKY transcription factor 39, and when inoculated with Xoo, the relative expression level of the resistant population was higher than that of the susceptible population. Resistance genes have previously been detected using only phenotypic change data. In this study, resistance candidate genes were detected using the concentration of metabolites produced in plants after inoculation with pathogens. This newly developed analysis method can be used to effectively detect and identify genes directly involved in disease resistance for future studies.

## 1. Introduction

Bacterial leaf blight (BLB) is caused by *Xanthomonas oryzae* pv. *oryzae* (Xoo) and is widely spread throughout Asia. This disease, Xoo, propagates around the vascular tissues, involving the water tube and phloem, and blocks the phloem, which prevents the movement of water and nutrients from the shoot and root, which causes the symptoms to appear [1]. Whitening of the leaves is one of the main symptoms of Xoo, and as a result, photosynthesis is hindered in plant leaves, which reduces both yield and quality [2]. Xoo, which causes BLB, has various races, of which K1, K2, K3, and K3a are reported to cause serious diseases in Asia, which accounts for 60% of the world’s rice yield [3,4]. Recent climate changes have also contributed to the steady increase in the incidence of mutations of Xoo—the pathogen causing BLB [5]. It is known that breeding disease-resistant varieties is the most effective response method, as the drug control effect is very low at present. Since BLB is a serious disease, breeding rice with BLB resistance using accurate estimation methods to identify BLB resistance genes is critical work [5,6]. However, cultivars with a single gene which is resistant to a single disease, if held in a specific area for a long time, will result in singularity of the variety, often leading to the breakdown of the resistance of the variety. Although many BLB-resistant rice cultivars exist, it is difficult to introduce these genes into *japonica* rice through hybridization. Therefore, quantitative trait locus (QTL) technology for the continuous screening of BLB resistance genes, aiming to identify a selection of new resistant varieties through molecular breeding technology, is the most effective way to treat this disease [7,8,9].

Molecular breeding technology is a key technology, allowing future agriculture to cope with the global food crisis. In molecular breeding technology, QTL analysis is an advanced major gene screening and mapping technology that can measure and locate the chromosomal location of target genes related to specific traits based on the corresponding gene map [10]. To achieve the screening’s purpose, it is necessary for the main resistance genes to undergo a molecular breeding process, and targeted variety improvements and development can be undertaken. In molecular breeding, this technology has a wide variety of applications, and in this study, we used different quantitative trait (visual inspection) data for related QTL analysis, meaning that our research utilizes phenotypic trait correlations. However, in the research on disease-resistant crop breeding and other disease-resistant breeding, the characteristics of diseased crops consist of internal tissue damage, followed by externally visible changes. Therefore, in this study, we utilized high-performance liquid chromatography (HPLC) to analyze the peak areas of the samples after inoculation and evaluated the quantitative characteristic data of the QTL analysis.

Once the plant undergoes a pathogen attack, the genes involved in various resistances are expressed by the plant, and are involved in various pathways and are expressed positively or negatively [11]. In particular, for disease resistance, the transcription factors or genes involved in signal transduction are actively regulated in the plant defense system [12]. Phytoalexin is a toxin produced by the toxin to counteract the pathogen attack. Phytoalexin has no negative effects on the plant itself, but acts as a toxin for the pathogens [13].

In this study, rice was inoculated with Xoo, which causes BLB, and the metabolites that were subsequently produced were analyzed using HPLC. In addition, QTL mapping was performed using the analyzed HPLC data, and any detected candidate genes involved in the generation of disease-resistant metabolites, such as phytoalexin, were screened in the detected region. The results of this study related to the genetic screening involved in the biosynthesis of phytoalexin and its related pathways has furthered the understanding of the mechanisms rice uses for resistance to various diseases, including white leaf blight. This will ultimately allow the quantity of rice produced to be increased.

## 2. Results

**Figure 1:**
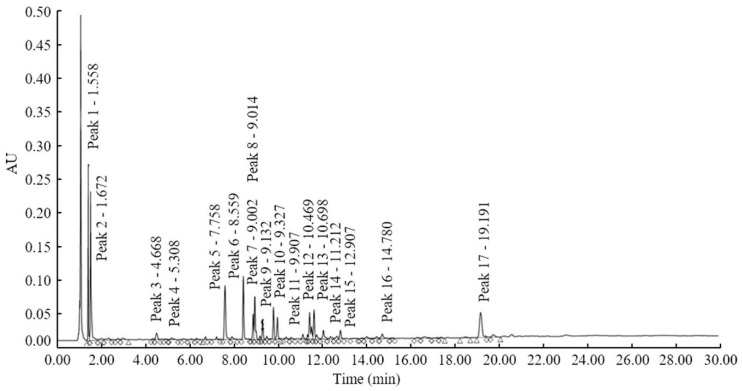
Analysis of the concentrations of resistant metabolites by means of high-performance liquid chromatography analysis after inoculation with *Xanthomonas oryzae* pv. *oryzae* (Xoo) in a 120 Cheongcheong/Nagdong double haploid (CNDH) population. A total of 17 metabolites were analyzed, and the concentration of metabolites produced after inoculation of the 120 CNDH population of Xoo varies according to the bacterial leaf blight (BLB)-resistant population and the susceptible population. Peak 1 is 1.558 min, Peak 2 is 1.672 min, Peak 3 is 4.668 min, Peak 4 is 0 min, Peak 5 is 7.758 min, Peak 6 is 8.559 min, Peak 7 was detected at 9.002 min, Peak 8 at 9.014 min, Peak 10 at 9.327 min, Peak 11 at 9.907 min, Peak 12 at 10.469 min, Peak 13 at 10.698 min, Peak 14 at 11.212 min, Peak 15 at 12.907 min, Peak 16 at 14.780 min, and Peak 17 at 19.191 min. Above the peak is the detected peak number and the detected time.

**Figure 2:**
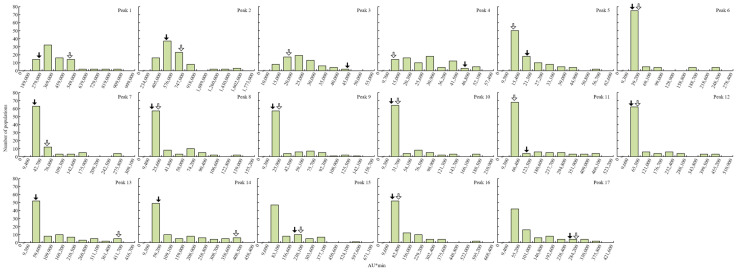
The normal distribution for high-performance liquid chromatography from Peak 1 to Peak 17. The concentrations of metabolites produced after *Xanthomonas oryzae* pv. *oryzae* (Xoo) inoculation in the 120 CNDH population are all normally distributed. Thus, these metabolites are the result of the interaction of one or more different genes. The black arrow represents Nagdong and the white arrow represents Cheongcheong.

HPLC analysis identified 17 peaks 14 days after Xoo inoculation in the 120 CNDH population (Figure 1 and Table S1). Peak 1 was detected at 1.558 min, Peak 2 at 1.672 min, Peak 3 at 4.668 min, Peak 4 at 5.308 min, Peak 5 at 7.758 min, Peak 6 at 8.524 min, Peak 7 at 9.002 min, 8 Peak 1 at 9.014 min, Peak 9 at 9.132 min, Peak 10 at 9.327 min, Peak 11 at 9.907 min, Peak 12 at 10.469 min, Peak 13 at 10.698 min, Peak 14 at 11.212 min, Peak 15 at 12.907 min, Peak 16 at 14.780 min, and Peak 17 at 19.191 min. All peaks were detected at the same time in the 120 CNDH population, with a different peak concentration observed for each population according to BLB resistance and sensitivity. The concentrations of Peak 1 of Cheongchoeng and Nagdong were 376,076 ± 124,025.0 and 498,711 ± 191,811.9, respectively. In the 120 CNDH population, the range of Peak value 1 is 189,497–908,957, with the average value of 3,767,570 ± 123,776.6. The concentrations of Cheongcheong and Nagdong for Peak 2 were 503,204 ± 1,659,503 and 732,059 ± 2,815,612, respectively, and the 120 CNDH population had concentration values in the range of 234,813–1,595,482, with an average value of 5,828,435 ± 2,297,990. The concentrations of Peak 3 of Cheongcheong and Nagdong were 30,903 ± 101,914 and 18,719 ± 71,996, respectively, with a concentration range in the 120 CNDH population of 9503–44,841, with an average of 232,257 ± 81,903. The mean of Cheongcheong and Nagdong for Peak 4 is 33,016 ± 108,883 and 10,607 ± 40,796, respectively, and the 120 CNDH population had concentration values in the range of 9784–52,081 and average values of 256,924 ± 109,873. The concentrations of Peak 5 for Cheongcheong and Nagdong were 15,481 ± 51,054 and 9939 ± 38,227, respectively, and the 120 CNDH population had concentration values of 9555–56,023, with averages of 172,034 ± 81,809. The concentrations of Cheongchoeng and Nagdong for Peak 6 were 20,033 ± 66,066 and 28,011 ± 107,735, respectively, and the 120 CNDH population had concentration values of 9353—248,110 and averaged 245,646 ± 321,734. The concentrations of Peak 7 in Cheongchoeng and Nagdong were 11,609 ± 38,285 and 63,311 ± 243,504, respectively, and the concentration of Peak 7 in the 120 CNDH population was in the range of 9499–274,272, with an average of 345,063 ± 475,518. The concentrations of Peak 8 of Cheongcheong and Nagdong were 76,965 ± 253,821 and 11,078 ± 42,608, respectively, and the concentration range of the 120 CNDH population was 9473–138,383, and the average was 286,421 ± 267,655. The means of Cheongcheong and Nagdong for Peak 9 were 20,081 ± 66,225 and 10,721 ± 41,235, respectively, and the 120 CNDH population had a concentration value in the range of 9351–141,933 and averages of 291,200 ± 288,064. The concentrations of Peak 10 of Cheongcheong and Nagdong were 22,800 ± 75,191 and 21,902 ± 84,238, respectively, and the 120 CNDH population had concentration values of 9385–188,335 and averaged 301,058 ± 361,585. The mean of Cheongcheong and Nagdong for Peak 11 was 13,378 ± 44,119 and 14,008 ± 53,877, respectively, and the 120 CNDH population had concentration values in the range of 9376–465,403, and the average was 608,898 ± 1,023,836. Peak 12 concentrations of Cheongcheong and Nagdong were 15,340 ± 50,589 and 19,239 ± 73,996, respectively, and the 120 CNDH population had concentration values of 9690–454,416 and averaged 633,335 ± 1,040,203. The concentrations of Cheongchoeng and Nagdong at Peak 13 were 16,510 ± 22,833 and 9830 ± 37,808, respectively, and the 120 CNDH population had 9355–411,198 concentration values, and the average was 801,537 ± 1,006,433. The concentrations of Peak 14 in Cheongchoeng and Nagdong are 23,246 ± 32,149 and 378,130 ± 1,454,346, respectively, and the concentration of Peak 14 in the 120 CNDH population was in the range of 9358–405,675, with an average of 1,037,657 ± 1,227,819. The concentrations of Peak 15 in Cheongchoeng and Nagdong were 56,108 ± 185,037 and 20,688 ± 79,569, respectively, and the concentration of Peak 15 in the 120 CNDH population was in the range of 9679–597,418, with an average of 948,998 ± 1,146,297. The concentrations of Peak 16 in Cheongcheong and Nagdong were 66,179 ± 218,250 and 19,052 ± 73,277, respectively, and the concentration range of the 120 CNDH population was 9621–594,881, and the average was 961,575 ± 1,082,567. The means of Cheongcheong and Nagdong for Peak 17 were 122,364 ± 40,354.1 and 280,222 ± 107,777.7, respectively, and the 120 CNDH population had concentration values in the range of 9409–372,965 and averaged 87,963.7 ± 92,997.7. For all HPLC analyses, the concentration values of all peaks showed continuous variation, with the majority of the peaks, including Peak 1, being skewed to the left in the normal distribution (Figure 2).

**Figure 3:**
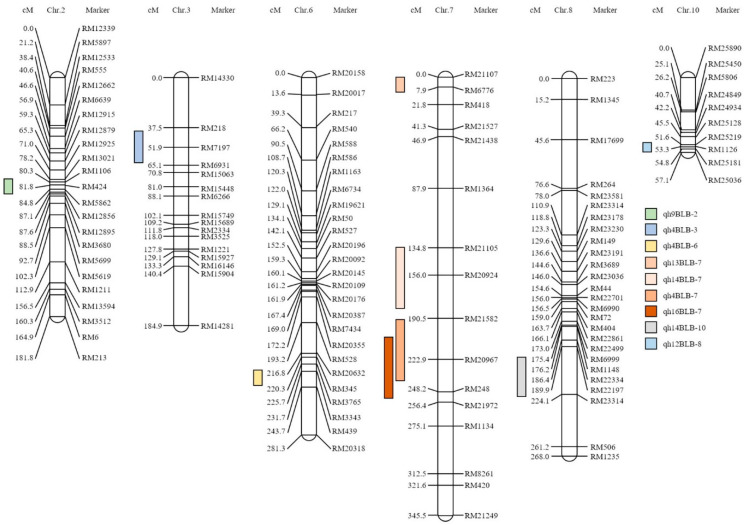
The chromosomal location of the quantitative trait locus (QTL), associated with high-performance liquid chromatography peak area data in the Cheongcheong/Nagdong double haploid genetic map. An LOD score of 3.0 or higher was detected when peak concentrations of 4, 9, 12, 13, 14, and 16 of the 17 metabolites were used. Only QTLs with an LOD score of 3.0 or higher were shown on the genetic map.

In addition, PCA was performed using the peak and BLB lesion lengths, which were detected using HPLC analysis. Peaks 4, 12, 13, 14, and 16 all had negative correlations with the lesion lengths (Figure S1 and Table S2). The QTL mapping analysis using the HPLC data of the 120 CNDH population, QTLs with an LOD score of 3.0 or higher were detected when Peaks 4, 9, 12, 13, 14, and 16 were used (Figure 3).

When using Peak 4, qh4BLB-3, qh4BLB-6, and qh4BLB-7 were detected. qh4BLB-3 was detected with an LOD score of 3.38 in RM7197–RM15063 of chromosome 3, and the explanatory phenotypic variation was 4.03% and was derived from the Cheongcheong allele. qh4BLB-6 was detected with an LOD score of 4.14 in RM345–RM439 of chromosome 6, with 12.09% of the phenotypic variation derived from the Nagdong allele. qh4BLB-7 was detected with an LOD score of 4.83 in RM21582–RM248 of chromosome 7, with a phenotypic variation of 3.93% that was derived from the Cheongcheong allele. qh9BLB-2 was detected in Peak 9 and was derived from a Nagdong allele, with an LOD score of 3.41 and an explainable phenotypic variation of 3.97% in RM1106–RM12856 of chromosome 2. Peak 12 was detected in qh12BLB-8 and RM22197–RM23314 of chromosome 8. The LOD score of qh12BLB-8 was 4.12, and it was possible to explain the phenotypic variation with 3.68%, which was derived from the allele of Nagdong. When using Peak 13, qh13BLB-7 was detected with an LOD score of 3.74 in RM21107–RM418 of chromosome 7. qh13BLB-7 can explain the phenotypic variation at 12.73%, and it was derived from the allele of Nagdong. In Peak 14, both qh14BLB-7 and qh14BLB-10 were detected. qh14BLB-7 was derived from RM21105–RM21582 of chromosome 7, with an LOD score of 10.89, explained a phenotypic variation of 20.22%, and the allele of Nagdong. qh14BLB-10 was detected with an LOD score of 3.03 in RM25219–RM25036 of chromosome 10. qh14BLB-10 was 1189% capable of explaining the phenotypic variation and was derived from the allele of Nagdong. In Peak 16, qh16BLB-7 was detected with an LOD score of 3.0 or higher and was derived from a LOD score of 3.76, with an explainable phenotypic variation of 2.51% and an allele of Nagdong in RM21582–RM248 of chromosome 7.

**Figure 4:**
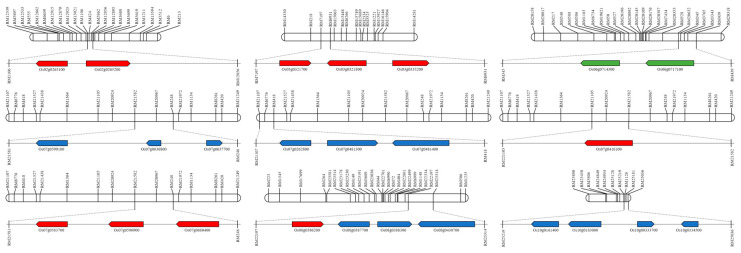
Physical map from the high-performance liquid chromatography data and the related quantitative trait locus (QTL) analysis using the Cheongcheong/Nagdong double haploid (CNDH) genetic map. Bacterial leaf blight resistance candidate genes around the marker area were screened based on the marker detected with an LOD score of 3.0 or higher. The chromosomal location of QTL was associated with the lesion length date of the CNDH genetic map. Red indicates the genes of the WRKY group; blue indicates disease resistance and plant defense group; green indicates the hormone signaling group.

The QTL mapping data allowed for the construction of a physical map (Figure 4): RM1106–RM12856 on chromosome 2, RM7197–RM15063 on chromosome 3, RM345–RM439 on chromosome 6, RM21582–RM248, RM21107–RM418, RM21105–RM21582 on chromosome 8, and RM22197–RM23314 on chromosome 10. In chromosome RM25219–RM25036 the open reading frames (ORFs) were searched to identify regions related to plant defense (Table S3). In these areas, we searched nine WRKY family genes, six plant defense genes, and three hormone signaling genes. In the WRKY family gene group, we detected a gene with a similar gene sequence to that of the WRKY transcription factor and a gene containing a DNA-binding WRKY domain. In the plant defense gene group, we also detected multidrug-resistance-associated proteins and disease resistance response proteins. In the hormone signaling group, we detected the senescence-associated family protein, mRNA splicing factor, and the auxin-responsive protein. After Xoo inoculation in the 120 CNDH population, the lesion length was analyzed.

**Figure 5:**
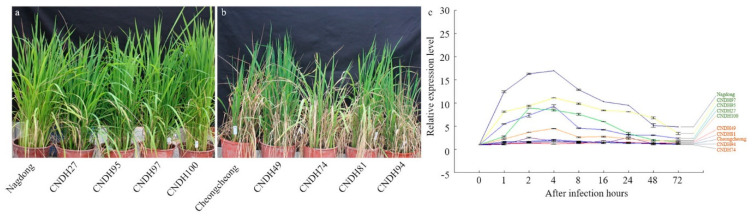
Quantitative RT-PCR analysis of the *OsWRKYq6* gene in high-resistance and weak-resistance varieties in the Cheongcheong/Nagdong double haploid (CNDH) population. (**a**) five groups (Nagdong, CNDH-27, CNDH-95, CNDH-97, and CNDH-100) of highly resistant populations selected from the field; (**b**) five groups (Cheongcheong, CNDH-49, CNDH-74, CNDH-81, and CNDH-94) of susceptible populations selected from the field. (**c**) quantitative RT-PCR analysis results of the defense gene, *OsWRKYq6*, in the resistant and susceptible groups in the CNDH population.

Finally, the BLB resistance groups included Nagdong, CNDH27, CNDH95, CNDH97, and CNDH100, and Cheongcheong, CNDH49, CNDH74, CNDH81, and CNDH94 were classified as BLB-susceptible groups. OsWRKY39 had a higher relative expression level at 1, 2, 4, 8, and 16 h after Xoo inoculation, with a significant difference at the 1% level in all resistant groups compared to the susceptible group (Figure 5).

## 3. Discussion

Across the world, BLB is a disease that causes serious damage to rice cultivars and causes the most damage to rice, among all bacterial diseases [14]. Xoo is a main contributor to the disease, and the pathogens multiply around the water duct and phloem, disrupting the movement of nutrients and water, and finally discoloring the leaves, causing a white color and disrupting photosynthesis. Therefore, the rice yields decrease and the grain quality is seriously affected [15].

As plants are immobile, they are constantly exposed to abiotic and biotic stress and must therefore protect themselves from attack from the various microorganisms with which they come into contact [16]. Plants require very sophisticated and special defense systems to survive [17]. Plants prevent the proliferation of pathogens by creating structural barriers to defend against pathogen attack or secreting harmful metabolites made through chemical reactions.

To date, to map the BLB resistance gene, QTL mapping was performed using major agricultural trait data that included lesion length after inoculation with the white leaf blight pathogen [18,19]. However, in this study, QTL mapping was performed using the concentration of secondary metabolites generated in plants using HPLC analysis after Xoo inoculation, which causes BLB. The CNDH population was used to map major QTLs that confer resistance to BLB. The 120 CNDH population was created by means of another F_1_ culture, created through crossing Cheongcheong and Nagdong to remove the isolated group and lines with irregular traits [20]. These populations have developed F_10_ in the current field, with specific characteristics in each population. At present, it is used as an intermediate model and is used effectively to test the function of genes and analyze the correlation between genes and phenotypes when identifying novel genes [21,22].

HPLC analysis after inoculation with the white leaf blight pathogen in the 120 CNDH population resulted in the detection of 17 peaks in each population, with a normal distribution for all observed or a left-skewed normal distribution. When analyzed by HPLC, numerous peaks were detected, in addition to 17 peaks. However, among the detected peaks, when BLB was induced in the 120 CNDH population, the concentration of the peak was changed and when the peaks that could be analyzed were finally selected, 17 peaks were detected as representatives. The peaks detected when BLB was induced are all important; however, in this research, the purpose is not to identify each of these substances, but the concentrations of these substances are different for BLB resistance and the sensitivity population. Therefore, we proposed a method to screen resistance genes using substances that cause differences. Therefore, all genes are involved in the quantitative traits expressed by various genes, as opposed to one single gene. The mapping of major QTLs confers BLB resistance using these data; QTLs were mapped on chromosomes 2, 3, 6, 7, 8, and 10. All the detected QTLs had an LOD score of 3.0 or higher, and among these QTLs, the highest explanatory phenotypic variation was 20.22%, and the lowest was 2.51%. In these areas, all ORFs related to plant defenses were evaluated. In the regions detected with an LOD score of 3.0 or higher, 50% of the WRKY family gene, 30% of the plant defense gene, and 20% of hormone signaling were searched.

When presented with environmental stress, the plant’s signal changes within its cells [23]. Specific stress-related genes are regulated at the transcription level, and secondary metabolites generated by either the upregulation or downregulation of transcription factors enhance the resistance of plants to stress [24]. The WRKY gene family is a conserved domain in various plants and acts on signal transduction in response to various abiotic and biotic stresses to give plants resistance [25]. In rice, over 100 WRKY family genes have been discovered to date [26]. Many WRKY genes exist in plants, as they cannot move, and defense genes have been developed in various ways to overcome various stresses in the immediate environment. *OsWRKYq6*, screened in this study, has a similar sequence to WRKY transcription factor 39 (data not shown). When candidate genes related to BLB resistance were screened by constructing QTL and physical mapping using the HPLC results, genes related to plant defense and hormone signaling were also evaluated, in addition to transcription factors. Among the plant defense genes, various pathogen-related (PR) genes were detected. These PR genes are expressed at the site on the plant where the pathogens attack, and play an important role in building the plant’s initial defense response system [27]. Additionally, in this study, hormone-related genes were screened. When plants are exposed to stress, plant hormones play a signaling role in the attempt to resist the stress. In particular, abscisic and salicylic acids are representative examples [28]. These hormones play a role in signaling the stress response of various pathogens and participate in physiological reactions, such as the aging of leaves and the dormancy of seeds, conferring resistance to plants [28]. Therefore, the BLB resistance candidate genes identified in this study are all important genes for the plant defense system.

Therefore, analyzing the concentration of plant defense metabolites that directly affect disease resistance and using this for QTL mapping can be effectively used to accurately map related genes. When the relative expression level of *OsWRKYq6* was analyzed following Xoo inoculation in the 120 CNDH population, which was used as an intermediate model, the BLB-resistant and susceptible populations were rarely expressed in the BLB-sensitive population, but they were observed in the resistant population. The relative expression level increased rapidly from the beginning of the inoculation. In comparison to several studies that identify WRKY transcription factor 39 as important in the early stages of the plant defense response, we suggest that the *OsWRKYq6* screened in this study is expected to be effectively used for the development of BLB-resistant rice cultivars and for the selection of resistant cultivars [29]. Currently, numerous studies related to BLB resistance have been reported, but QTLs mapped at the same location as these are very rare [30]. The reason for this is that the type of group used in each study, the number of groups, the genetic background, and environmental factors acted differently [31]. To map the BLB resistance gene, the concentration of secondary metabolites produced in plants after Xoo inoculation in a 120 CNDH population was used directly in this study. Normal QTL mapping uses phenotypic data such as lesion length, resistance score, and plant length; however, in this study, as the concentration of secondary metabolites are produced when pathogens attack the plants, it is difficult to synthesize disease-resistant metabolites. Nevertheless, the mapping of the genes that were directly involved was possible.

*OsWRKYq6*, which was detected and identified in this study, can be effectively used to investigate the function of disease-resistant metabolites in plants through the mass production of metabolites and to breed white leaf blight-resistant rice varieties. We have demonstrated that it can be actively used for resistance studies.

## 4. Materials and Methods

### 4.1. Rice Materials and Field Design

The materials of the Cheongcheong/Nagdong double haploid (CNDH) population were planted in a field at Kyungpook National University from 2010. An F1 was obtained through the crossing of Cheongcheong (Indica) with Nagdong (Japonica) and cultured to doubled haploids, and the CNDH 120 line was developed for genetic mapping. A seed disinfectant was used to sterilize each of the 120 seeds of the CNDH population and inoculated in darkness at 33.3°C for 3 days. This was sown in the field of Kyungpook National University; 5 rows and 125 plants were transplanted, with 25 plants per row per population with a planting distance of 30 × 15 cm. The amount of fertilization was 9, 4.5, and 5.7 kg/10a (N, P_2_O_5_, K_2_O, respectively) and cultivated according to the Agricultural Science and Technology Research Survey Standard of Rural Development Administration.

### 4.2. K3 Strain Culture Media, Inoculation, and Infection Assessment

The experimental inoculation was conducted using the K3 strain material. The K3 strain of Xoo was cultured on potato sucrose agar media (PSA) containing peptone (10 g), sucrose (10 g), and agar (12 g) [32]. Prior to inoculation, the K3 strain was grown in liquid PSA for 72 h at 27 °C. The inoculation was started 40 days after the transplantation of the rice seedlings. The center 10 plants in each row were inoculated, and 5 leaves per plant were inoculated. A pair of scissors was dipped into the K3 suspension and subsequently used to cut the leaf tip at approximately 3–4 cm for inoculating the bacterial strain. After the inoculation of the pathogenic strain, the lesion length of the infected leaf was measured after 14 days. The lesion length analysis measured the advancement of the pathogen from the inoculation site and calculated the mean and standard deviation.

### 4.3. Extraction of the Infected Rice Leaf Components and HPLC Analysis

Inoculated leaves from the CNDH population were sampled on the 14th day after Xoo inoculation. A total of 5 g of stem and leaf powder was cut and placed into a falcon tube containing a solution of 50 mL of 90% methanol in water for 3 days in dark conditions. Samples were subsequently sonicated at 24 ± 1 °C for 20 min. The methanol solution was then treated three times with hexane. Subsequently, the methanol solution was dissolved in water and evaporated. The dry sample was eluted in 1 mL with a filtration size of 0.45 mL. The HPLC-conditioned methanol elute was separated using a reverse-phase HPLC column (ZORBAX 4.6 250 10 mm, particle size 5 mm, Agilent, Santa Clara, CA, USA).

### 4.4. QTL Analysis

QTL analysis was performed using WinQTLcart version 2.5 software, which analyzed the BLB lesion length of the CNDH population. The genetic mapping of the CNDH 120 line, with an average distance of 10.6 cM, was constructed using Mapmaker version 3.0 with 222 SSR markers. The values of the metabolite concentrations were analyzed in the Kosambi Function using the Composite Interval Mapping method and a threshold LOD value of 3.0 or higher [33]. The identified QTLs were named following the method adapted by McCouch et al. (2008) [34]. Several factors were required for the program, such as marker names, the genetic distance for each marker, chromosome number, genotyping data, and values of the target traits.

### 4.5. RNA Extraction

Total RNA from rice leaf samples was extracted using the RNeasy Plant Mini Kit (QIAGEN, Cat. 74904, Hilden, Germany) at different time points (0 h, 1 h, 2 h, 4 h, 8 h, 16 h, 24 h, 48 h, and 72 h) after inoculation. Samples obtained at each inoculation timepoint were immediately placed in liquid nitrogen and manually ground using a mortar and pestle. According to the manufacturer’s instructions for the RNeasy Plant Mini Kit, the ground rice powder was suspended in 450 µL of Buffer RLT with β-mercaptoethanol and vortexed. The lysate was transferred to a QIAshredder spin column, centrifuged at 13,000 rpm for 1 min, and then transferred into a fresh tube. Next, a 0.5 volume of ethanol (96–100%) was added. The mixture was transferred to an RNeasy spin column and centrifuged for 15 s at 10,000 rpm, and the wash-through was discarded. The column was washed by adding 700 µL of Buffer RW1 and centrifuged for 1 min at 10,000 rpm. Finally, 500 µL Buffer RPE was added to the column, centrifuged at 10,000 rpm for 1 min, and the wash-through was discarded. This step was repeated once more, with centrifugation for 2 min. The RNA was eluted by adding 40 µL RNase-free water onto the column and it was centrifuged for 2 min at 13,000 rpm.

### 4.6. cDNA Synthesis

Diluted RNA from the rice leaf that was collected at different time periods after inoculation was used to synthesize cDNA for quantitative RT-PCR analysis. Master mixes were composed of 4 µL of 5× cDNA synthesis mix (SuperScript IV One-Step RT-PCR System, Thermo Fisher, Cat. 12594025, MA, USA), 10 µL of diluted RNA, 5 µL of RNase-free water, and 20× RTase (enzyme). The master mix was incubated for 30 min at 42 °C. After incubation, the cDNA concentration was measured using a NanoDrop 2000 spectrophotometer (Thermo Scientific, Wilmington, DE, USA).

### 4.7. Quantitative RT-PCR Analyses

For first-strand cDNA synthesis, the qPCRBIO cDNA synthesis kit was used with a 400-ng input of the total RNA. Quantitative RT-PCR was performed using the Eco Real-Time PCR system (Illumina, Inc., San Diego, CA, USA), 2X qPCRBIO SyGreen (www.pcrbio.com (accessed on 13 September 2020), London, UK), and primers for the genes of interest. The simple sequence repeat marker primers were used to determine WRKY transcription factor 39 (forward 5′-ATGGACGGCGACGCATG-3′, reverse 5′-TCATCTCCACTCGTAGCTC-3′). Among the CNDH populations, BLB resistance lines (OXCM, Nagdong, CNDH27, CNDH95, CNDH97, and CNDH100) and susceptible lines (Cheongcheong, CNDH49, CNDH75, CNDH81, and CNDH94) were compared to the expression levels of WRKY transcription factor 39 after inoculation with the K3 strain of Xoo. The relative gene expression levels of WRKY transcription factor 39 were compared and analyzed in the CNDH populations.

### 4.8. Statistical Analysis

Correlations between the peak area in the HPLC analysis and infection length data of the corresponding leaf samples were assessed using the principal component analysis (PCA) statistical model in the XLSTAT software. To extract materials from each 120 CNDH population, 125 plants were planted in each population, and 5 plants were randomly selected from among them. The mean and standard deviation of the lesion lengths and HPLC peak area data of five plants were statistically analyzed using SPSS [33]. All experimental data were replicated three times.

## 5. Conclusions

BLB is an important and devastating rice disease (Xoo) caused by the pathogen *Xanthomonas oryzae* pv. In recent years, the continuous deterioration of the climate and global warming has provided good growth conditions for BLB, leading to a continuous increase in the incidence of BLB on a global scale. The identification of new resistance genes of BLB and the cultivation of resistant varieties is now of paramount importance. QTL is one of the main auxiliary technologies of molecular breeding. In this study, we used the peak data from HPLC analysis to replace the quantitative trait (visual) data commonly used in QTL. The results showed that such a method is effective and feasible. Therefore, a variety of analysis methods can be used to replace the traditional quantitative trait data to obtain the associated target gene interval through QTL analysis. This greatly expands the breadth of trait research for future resistance-breeding research. At the same time, the CNDH population used in this study had a unique phenotype, which was attributed to the gene fixation of many generations. This enables QTL to more accurately analyze and locate the relationship between phenotypes and genes in CNDH-based gene mapping analysis.

## Data Availability

The datasets obtained or analyzed during the current study are available from the corresponding author on reasonable request.

## Supplementary Materials

The following are available online at https://www.mdpi.com/article/10.3390/plants10102145/s1, Figure S1: PCA (Principal Component Analysis) statistical analysis between the peak area in the HPLC analysis results and the infection length data of the corresponding leaf samples. Table S1: The plant traits and peak no. of 120 CNDH (Cheongcheong/Nagdong double haploid) populations by HPLC (high-performance liquid chromatography). Table S2: Details of QTL mapping using HPLC analysis results of 120 CNDH population after *Xanthomonas oryzae* pv. *oryzae* inoculation. Table S3: Candidate genes involved in Bacterial leaf blight resistance.
