# Supplementary material for: Applying HPLC to Screening QTLs for BLB Resistance in Rice
Source: Plants (Basel). 2021 Oct 9;10(10):2145. doi: 10.3390/plants10102145 (PMC8537431; doi:10.3390/plants10102145)
Supplement: Supplementary file 1 [file plants-10-02145-s001.zip › Plants_supplementary materials.pdf]

Supplementary Materials

Table S1. The plant traits and peak no. of 120 CNDH (Cheongcheong/Nagdong double haploid) populations by HPLC (high-performance liquid chromatography).

Peak No.	Parents		CNDH population	
	Cheongcheong	Nagdong	Range	Means
1	376076±124025.0	498711±191811.9	189497-908957	376757.0±123776.6
2	503204±165950.3	732059±281561.2	234813-1595482	582843.5±229799.0
3	30903±10191.4	18719±7199.6	9503-44841	23225.7±8190.3
4	33016±10888.3	10607±4079.6	9784-52081	25692.4±10987.3
5	15481±5105.4	9939±3822.7	9555-56023	17203.4±8180.9
6	20033±6606.6	28011±10773.5	9353-248110	24564.6±32173.4
7	11609±3828.5	63311±24350.4	9499-274272	34506.3±47551.8
8	76965±25382.1	11078±4260.8	9473-138383	28642.1±26765.5
9	20081±6622.5	10721±4123.5	9351-141933	29120.0±28806.4
10	22800±7519.1	21902±8423.8	9385-188335	30105.8±36158.5
11	13378±4411.9	14008±5387.7	9376-465403	60889.8±102383.6
12	15340±5058.9	19239±7399.6	9690-454416	63333.5±104020.3
13	16510±2283.3	9830±3780.8	9355-411198	80153.7±100643.3
14	23246±3214.9	378130±145434.6	9358-405675	103765.7±122781.9
15	56108±18503.7	20688±7956.9	9679-597418	94899.8±114629.7
16	66179±21825.0	19052±7327.7	9621-594881	96157.5±108256.7
17	122364±40354.1	280222±107777.7	9409-372965	87963.7±92997.7

Table S2. Details of QTL mapping using HPLC analysis results of 120 CNDH population after *Xanthomonas oryzae* pv. *oryzae* inoculation.

Peak No.	QTLs	Interval Markers[z]	Chromosome	LOD	Additive effect[y]	R^{2x}	Increasing effects[w]
4	qh4BLB-3	RM7197-RM15063	3	3.38	4.03	1.62	Cheongcheong
	qh4BLB-6	RM345-RM439	6	4.14	12.09	4.12	Nagdong
	qh4BLB-7	RM21582-RM248	7	4.83	3.93	1.13	Cheongcheong
9	qh9BLB-2	RM1106-RM12856	2	3.41	3.97	1.53	Nagdong
12	qh12BLB-8	RM22197-RM23314	8	4.12	3.68	1.42	Nagdong
13	qh13BLB-7	RM21107-RM418	7	3.74	12.73	4.34	Nagdong
14	qh14BLB-7	RM21105-RM21582	7	10.89	20.22	7.46	Nagdong
	qh14BLB-10	RM25219-RM25036	10	3.03	11.89	3.71	Nagdong
16	qh16BLB-7	RM21582-RM248	7	3.76	2.51	0.62	Nagdong

[z] Interval markers are those within the significance threshold on each border of the QTL range

[y] The proportion of evaluated phenotypic change attributable to a particular QTL was estimated using the coefficient of determination (R^2)

[x] Positive values of the additive effect indicate that alleles from Cheongcheong are in the direction of increasing the traits

[w] Increase allele is the source of the allele that causes an increase in the measured trait.

Table S3. Candidate genes involved in Bacterial leaf blight resistance.

Item	Gene	No. of genes
WRKY family	Similar to WRKY transcription factor 39.	9
	WRKY transcription factor 39.	
	WRKY transcription factor 78.	
	Similar to SUSIBA2-like (WRKY transcription factor 80).	
	DNA-binding WRKY domain containing protein.	
	Similar to WRKY transcription factor 53	
	WRKY transcription factor 69.	
	Similar to WRKY transcription factor 55.	
	Similar to WRKY1 (WRKY transcription factor 17).	
Plant defense	Similar to Multidrug resistance associated protein 2.	6
	Plant disease resistance response protein family protein.	
	Similar to Resistance protein candidate (Fragment).	
	Disease resistance protein family protein.	
	Similar to UVB-resistance protein-like.	
	Similar to Resistance protein candidate (Fragment).	
Hormone signaling	Senescence-associated family protein.	3
	mRNA splicing factor SYF2 family protein.	
	Auxin responsive SAUR protein family protein.	

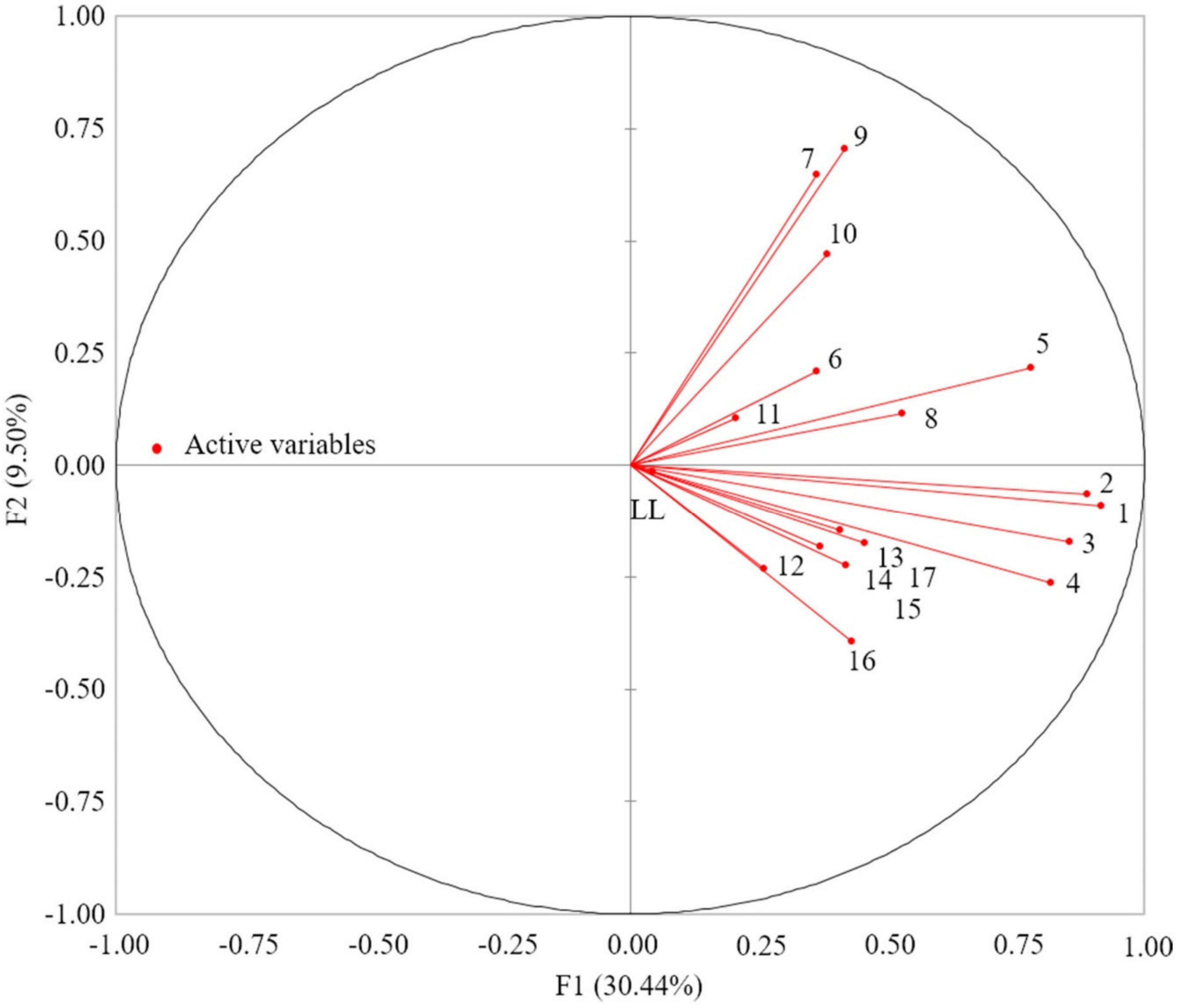

Figure S1. PCA (Principal Component Analysis) statistical analysis between the peak area in the HPLC analysis results and the infection length data of the corresponding leaf samples. It shows a projection of the initial variables in the factors space. When two variables are far from the center, then, if they are: Close to each other, they are significantly positively correlated (r close to 1); If they are orthogonal, they are not correlated (r close to 0). Number means peak number. LL, Lesion length.
